# Circulating Interleukin-6 (but Not Other Immune Mediators) Associates with Criteria for Fried's Frailty among Very Old Adults

**DOI:** 10.1155/2020/6831791

**Published:** 2020-11-04

**Authors:** Gilberto Santos Morais Junior, Diego Ignacio Valenzuela Perez, Audrey Cecília Tonet-Furioso, Lucy Gomes, Karla Helena Coelho Vilaça, Vicente Paulo Alves, Clayton Franco Moraes, Otávio Toledo Nóbrega

**Affiliations:** ^1^Health Faculty, Campus Universitário Darcy Ribeiro, Universidade de Brasília, Brasília, DF, Brazil; ^2^Kinesiology School and Physical Activity and Sports Science Master Program, Universidad Santo Tomás, Puerto Montt, Chile; ^3^Graduate Program in Gerontology, Campus Taguatinga, Universidade Católica de Brasília, Brasília, DF, Brazil; ^4^Division of Geriatric Medicine, Department of Medicine, McGill University, 1001 Boul Décarie, Montreal, QC, Canada

## Abstract

**Methods:**

One hundred and sixty-one very old patients (aged ≥80 years) devoid of cognitive decline were eligible for analyses. Clinical and biochemical data along with physical and cognitive assessments encompassing dual-energy X-ray scans and hand dynamometry were adopted to investigate frailty criteria, while circulating immune mediators (*IFNγ, IL-2, IL-4, IL-6, IL-10,* and *TNFα*) were assessed using high-throughput flow cytometry.

**Results:**

Preliminarily, *IL-6* correlated positively with waist-to-hip ratio and C-reactive protein and negatively with glycemia. In analyses controlled for these factors, serum levels of IL-6 were comparatively augmented among the very old participants with reduced grip strength (OR = 3.299; 95% CI 1.08–6.09; *p*=0.032) and among those with slow walk speed (OR = 2.460; 95% CI 1.16–7.05; *p*=0.022).

**Conclusions:**

Our study shows a strong negative correlation of *IL-6* levels with Fried's frailty components of grip strength and walk speed in very old adults, regardless of confounding factors.

## 1. Introduction

The aging process is responsible for dysfunctions in skeletal muscles, strength, and bone mass and increases the risk of disability *especially among very old adults* [1–3]. Frailty is a geriatric condition resulting from physiological changes that affect the musculoskeletal, immune, and neuroendocrine systems [4], leading to functional decline in elderly population and increasing prevalence of chronic degenerative diseases, depression, and mortality [5–7].

Studies point to a greater risk of negative events in terms of health and functionality among the very old (≥80 years) subset of older individuals with the syndrome, with evidence that frailty in this stratum contributes to increased mortality related to falls [8, 9]. Despite that, the fundamental characteristics of frailty are well established on the basis of declines in lean body composition as well as loss in muscular endurance and performance, and the pathophysiological changes that underlie this clinical entity are still not well understood. In this context, a process entitled as “inflammageing” that usually refers to slightly, subclinically higher levels of serum immune mediators in older adults relative to youngsters, both groups devoid of overt infectious disease, is associated with the pathophysiology of chronic conditions such as frailty [10–12] and susceptibility to infectious illnesses such as COVID-19 [13]. *Enhanced levels of circulating immune mediators such as IL-6 are often taken as a surrogate for “inflammageing” and are consistently associated with the frail phenotype and mortality* [10, 14].

A proinflammatory context is understood as a strong contributor to the onset of frailty [15–17], and mediators such as *interferon gamma (IFNγ)* and *tumor necrosis factor alpha (TNFα)* have already been implicated with the development of the syndrome [17, 18]. There is evidence that anti-inflammatory interleukins (ILs) such as *IL-10* and *IL-4*, although implicated to a lesser extent, also associate with the *frailty phenotype* [19–21]. However, among all known mediators, *IL-6* appears to play a prominent role in pathophysiology of the condition [16, 22], with direct evidence in favor of high levels of the mediator as well as of C-reactive protein (C-RP) in the onset of frailty [23–25], possibly due to its extra-immune effects on protein degradation and related metabolic pathways [26].

Each isolate component of frailty *per se* represents a domain of importance to the overall health and functionality of the oldest old. Hand grip strength, for instance, is an important marker of morbidity and mortality [27], and series of reports have demonstrated that low performance correlates with higher levels of subclinical inflammation [28–30]. Also, other reports have associated augmented serum concentrations of *IL-6* with lower gait speed and reduced muscle strength in a context of frailty [31–34]. Thus, the present study aimed to investigate a possible association of frailty components (as well as of the phenotype itself) with total serum levels of a broad, comprehensive panel of inflammatory mediators evaluated in a sample of very old Brazilian patients.

## 2. Materials and Methods

### 2.1. Study Design

The present study has a cross-sectional analytical nature and has enrolled very old frail and nonfrail participants living in Brasília, Federal District, Brazil.

### 2.2. Sample

**Figure 1:**
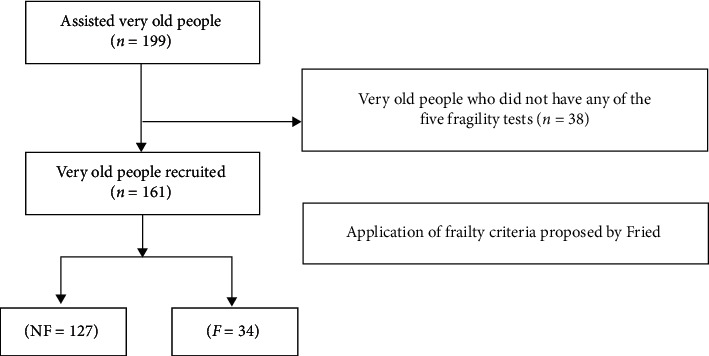
A schematic drawing of the sample. NF = nonfrail and *F* = frail.

A sample of 161 elderly, nondemented individuals aged ≥80 years was constituted based on patients that regularly and electively attended health care services for older adults in the Brazilian Federal District, Brazil (Figure 1). The elderly were characterized as carriers or not of frailty based on the criteria developed by Fried et al. [4]. All participants signed a free and informed consent form to participate in the research. Inclusion criteria consisted of being 80 years of age or older and not having mobility or cognitive problems. Exclusion criteria were based on recommendations by Ferruci et al. [35], namely, having important cognitive deficits (including Parkinson's or Alzheimer's diseases), detectable sensory deficit (especially in vision), active clinical condition with immunological impact (infection, autoimmunity, or malignancy), and regular use of immunomodulatory drugs in the last 30 days. Clinical evaluation of each patient was according to basic procedures of semiology and semiotics, with diagnosis of the most prevalent clinical conditions performed according to guidelines of specialized societies taking into account the history of diseases and consumption of drugs as described in medical records or self-reported [36]. Cognitive decline was investigated based on criteria of the American Psychiatric Association (APA/DSM-IV) and of the Alzheimer's Disease Assessment Scale-Cognitive Subscale (ADAS-Cog) for investigation of suspected cases of dementia [37]. This study followed the recommendations of the Declaration of Helsinki and was approved by the Research Ethics Committee of Catholic University of Brasilia, under registry number 50075215.2.0000.0029.

### 2.3. Blood Collection

Initially, blood drawn by venipuncture was obtained by trained nursing professionals, yielding serum samples by centrifugation at 2,500 rpm for 15 min that were processed immediately for clinical biochemistry by one same laboratory. For the analysis of the inflammatory panel, serum samples were quickly stored at −80° for later evaluations.

### 2.4. Anthropometric and Biochemical Measures

The anthropometric measurements obtained consisted of body mass (kg) and height (m) as well as circumferences of the waist (WC) and hip (QC), with which body mass indexes (BMI) and waist-to-hip (W-H) ratios were calculated based on WHO recommendations [38]. Measurements of body fat percentage (%*F*) and bone mineral density (BMD) were obtained with double-energy X-ray absorptiometry (DXA) and model DPX-IQ (GE Lunar Corporation) of the pencil beam type operated according to manufacturer's recommendations with data analyzed with software version 4.7.

Clinical biochemistry analyses were performed following laboratory protocols and quality control steps compatible with routine practices. Levels of glucose, triglycerides, total cholesterol, and fractions were measured by enzymatic, kinetic, or colorimetric tests (as appropriate) with reagents compatible with the HumanStar 600 equipment (InVitro^®^). LDL was estimated according to Martin's formula [39]. The same automatized platform was used to determine levels of ultrasensitive C-reactive protein and glycated hemoglobin by turbidimetry and ion-exchange high-performance liquid chromatography, respectively.

### 2.5. Frailty Characterization

Physical activity was measured by self-report using a translated version of the Minnesota Leisure Time Activities Questionnaire, validated for the Brazilian Portuguese [40]. Exhaustion was also measured by self-report using items 7 and 20 of the Center for Epidemiological Studies Scale for Depression-CES-D [41]. The hand grip strength was measured using a hydraulic dynamometer (Jamar brand, Model 5030J1, Lafayette Instruments Inc.) through three runs with one minute of rest between attempts and a final score determined as average of all attempts [42]. Finally, walking time was measured using the 4.6 m test, based on recommendations of Guralnik et al. [43], with the final score as an average of three runs [44].

Frailty was defined based on the criteria proposed by Fried et al. [4] and adapted by the Network of Studies on Frailty in Elderly Brazilians (FIBRA) [44, 45], comprising five stages. Weight loss was defined as a reduction of ≥4.5 kg or ≥5% of body weight in the last year. Exhaustion was excessive lack of fitness to perform tasks of daily living, with a participant unable to perform tasks for more than three consecutive days. Women scored for low activity if showing weekly energy expenditure <270 kcal, whereas men if with weekly energy expenditure <383 kcal. Participants who performed among the 20% lower levels of grip strength and among those within the 20% with higher values of *walking time* (adjusted for sex, BMI, or height, as appropriate) scored for frailty.

### 2.6. Inflammatory Profile

Inflammatory profile was assessed using high-throughput flow cytometry (FACS Verse model; BD Biosciences, San Jose, CA, USA) with the serum previously collected and the Human Th1/Th2 cytokine kit as reagent (BD Biosciences) to assess six mediators: *IFNγ, IL-2, IL-4, IL-6, IL-10,* and *TNFα.* The reactions were performed following the manufacturer's protocol, producing a titration curve with standards provided by the kit. All scores were estimated by interpolation of the respective curve. Whenever a given sample yielded out of range of outlying readings, the assay was repeated with an original or diluted sample (as necessary) until a minimum of three hundred events were acquired for each type of cytokine bead used. All data were analyzed using FCAP software, version 3.0 (BD Biosciences).

### 2.7. Statistical Analysis

Initially, the Kolmogorov–Smirnov test was performed to observe data distribution. *Student's t test for independent samples was used for anthropometric data, whereas the nonparametric Mann–Whitney test was used for biochemical data and inflammatory profile, and the chi-square test was employed for categorical variables. Data with normal distribution are expressed as mean and standard deviation, while data with non-normalized dispersion are shown as median and interquartile range, and categorical data are indicated in proportions*. After initial analyses, a logarithmic transformation of the inflammatory profile was performed, followed by Pearson's correlation analyses between these log-transformed inflammatory measures and scores of clinical/biochemical characteristics and of each component of the frailty syndrome. Univariate and multivariate analyses were performed by binary logistic regression, with the aim of analyzing association of isolate frailty components (as well as of the phenotype as a whole) with each immune marker. For construction of a multivariate model, variables that obtained *p* < 0.20 in univariate analysis were considered. All analyses were performed using SPSS Software, version 22.

## 3. Results

Of the 161 very old adults, 127 were characterized as nonfrail while 34 were identified as carriers of the syndrome (Figure 1). Anthropometric, biochemical, and clinical characteristics are shown in Table 1, according to the frailty status. There was no significant difference in anthropometric and biochemical profile between groups.

To assess which clinical and metabolic conditions of the sample could interfere with main models of association between inflammatory mediators and frailty components, correlation tests were run with the circulating levels of each cytokine across the anthropometric measures as well as the biochemical variables. In these tests, *IL-6* showed a positive correlation with *W-R ratio* and with levels of *C-RP*, as well as a negative correlation with glycemia. No other cytokine was found to correlate with the clinical traits assessed (Table 2).

To estimate to which proportion inflammatory mediators could be found in unequal quantities according to each component of Fried's frailty (or to the phenotype as a whole), univariate models of binary logistic regression were tested. In these calculations, covariates that showed a previous correlation with *IL-6* (Table 2) were included *so as* to *control analyses* for confounding variables *(W-H ratio, glucose,* and *C-RP).* With this approach, only the grip strength component was associated with the variables included in the model [*χ*^2^(4)=12.217; *p*=0.016, *R*^2^ = 0.134]. As a result, the very old participants with a level of strength compatible with frailty showed an almost *4.7-fold* greater chance of having *IL-6* titers higher than those found *with the same trait within nonfrail limits,* regardless of the associations of *W-H ratio*, glycemia, and C-RP with levels of the cytokine (Table 3). According to this univariate approach, no other component of the frailty syndrome (nor the entire phenotype) was associated with inflammatory variables.

As confirmatory analysis, a multivariate model was tested with variables to which *p* < 0.20 was obtained (C-RP and *IL-6* for grip strength and only *IL-6* for *walking time*), and it was verified that elderly people with reduced grip strength were 3 times more likely of presenting augmented levels of *IL-6* [*χ*^2^(2)=8.214; *p*=0.016, *R*^2^ = 0.085] when compared to the nonfrail participants (OR = 3.299; 95% CI 1.08–6.09; *p*=0.014), while the same was not observed for C-RP (OR = 0.734; 95% CI 0.51–1.07; *p*=0.107). Elderly people with *high walking time* were 2.5 times more likely [*χ*^2^(1)=5.897; *p*=0.015, *R*^2^ = 0.060] to have circulating *IL-6* at levels significantly higher than those found in the very old nonfrail examined participants (OR = 2.460; 95% CI 1.16–7.05; *p*=0.022).

## 4. Discussion

The present study aimed to investigate the individual association of each component of Fried's frailty syndrome (and of the phenotype as one) with total serum levels of a set of inflammatory mediators evaluated in a sample of very old Brazilian patients devoid of major cognitive decline cases. As an initial result, an exploratory analysis revealed that only reduced scores of grip strength associated with higher levels of serum *IL-6*. Confirmatory analyses not only reassured the negative correlation between *IL-6* and hand grip strength (regardless of covariates) but also revealed a similar relationship between *walking time* scores and the mediator.

Inflammatory mediators emerged as important surrogate markers for performance in traits linked to the skeletal muscle during the aging process [46–50], with evidence for a strong association of supraphysiological concentrations of *IL-6* with loss of physical functioning [51]. Interestingly, these findings are independent either of current use of anti-inflammatory drugs or of other comorbidities [51]. It no longer surprises that age-related chronic conditions are often characterized by a low-grade augmentation of circulating titers of proinflammatory mediators, which includes *IL-6* [52]. But, it remains debatable to which phenotype(s) these idiopathic imbalances in systemic mediators impact the most.

In line, the mechanisms involved in declines of strength and integrity of the skeletal muscle with age and participation of immune signals are still not well understood [53]. Reports repeatedly demonstrate that myofiber atrophy comes from reduced protein synthesis and exacerbated protein catabolism, the latter mainly due to cytoplasmic (ubiquitin-proteosome system) and vesicular (autophagy) protein degradation mechanisms [54, 55]. So far, some evidence points to proinflammatory interleukins as regulators of these pathways [56–61].

There is growing evidence that chronic *IL-6* exposure directly stimulates muscle wasting by activating the ubiquitin-proteosome pathway, which in turn destroys cytosolic and nuclear proteins in fiber cells [58, 59], with implications for physical performance. Moreover, evidence shows that *IL-6* action on muscle includes activation of the *signal transducer and activator of transcription 3 (STAT3)* [62–64], a fundamental autophagy regulator stored in the cytosol whose translocation to the cell nucleus occurs upon phosphorization to bind to specific DNA elements. STAT3 is known to activate proautophagic effector genes as those for the protein-interacting protein 3 (BNIP3) and the hypoxia-inducible factor (HIF1A) [65, 66] and other downstream regulators of the autophagic process such as the transcription factors FOXO1 and FOXO3 [67, 68] which in turn increases expression of a myriad of other genes that execute autophagy in the cell. Furthermore, there is evidence that *IL-6* stimulates the adrenal-pituitary axis, causing release of ACTH that increases circulating levels of cortisol, impairing synthesis in the striated muscles [56, 57]. Thus, our finding corroborates the hypothesis that even slightly augmented levels of *IL-6* can possibly produce effects that predispose to the physical phenotype of frailty.

The authors do not intend to drive the readers' sight away from studies with an approach similar to ours or from the fact that our findings resemble those reported elsewhere. On our behalf, our report places a contribution by specifically investigating the subset of low-functioning very old adults, while most of the literature that evaluates immune mediators (intended as biomarkers for frailty) across performance levels simply addresses older individuals in general and usually includes high-functioning individuals [61, 69]. Regardless that the physical performance scores herein assessed were statistically different across frail and nonfrail individuals, it is noteworthy that mean measures were far from different in terms of clinical relevance. Accordingly, no single score of hand grip strength or walking time was greater than 25 kgf or lower than 4 s, respectively, which is compatible with an overall status of poor physical functioning.

Despite the cross-sectional design and the limited sampling, this study has strengths in the sense that some level of homogeneity was possible when recruiting participants for analyses, with all very old adults devoid of major cognitive impairments, so to rule out possible disabilities owing to cases of dementia. Moreover, it was possible to detect and control covariates related to *IL-6* prior to obtaining the main outputs described herein, allowing to assume that spurious associations were unlikely in the scenario. At last, assessing at once several mediators that notably demonstrate extraimmunological effects would reduce partialities related to addressing “favorite players” only, also known as the positive publication bias around IL-6.

## 5. Conclusions

Our study shows a strong negative association of circulating levels of *IL-6* with levels of hand grip strength and of walking time assessed according to Fried's frailty phenotype among very old individuals.

## Figures and Tables

**Table 1:** Anthropometric and metabolic characteristics of the sample according to clinical condition.

	*Nonfrailty* (*n* = 127)	*Frailty* (*n* = 34)	*p*
Age (years)^*∗*^	84.3 ± 4.1	84.5 ± 4.0	0.800
Body mass (Kg)^*∗*^	64.0 ± 12.7	62.9 ± 11.6	0.663
Height (m)^*∗*^	1.6 ± 0.1	1.5 ± 0.1	0.314
BMI (kg.m^−2^)^*∗*^	26.1 ± 4.6	26.3 ± 4.0	0.748
Body fat (%)^*∗*^	32.6 ± 9.9	32.7 ± 9.1	0.975
WC (cm)^*∗*^	92.9 ± 12.2	95.4 ± 12.7	0.338
HC (cm)^*∗*^	99.9 ± 9.7	99.8 ± 9.2	0.967
W-H ratio ^*∗*^	0.9 ± 0.1	1.0 ± 0.1	0.128
MMSE (score)^*∗*^	20.7 ± 6.5	18.5 ± 6.3	0.087
Glucose (mg/dL)^*∗∗*^	93.0 (87.0–105.0)	94.0 (78.5–105.5)	0.541
TC (mg.dl^−1^)^*∗∗*^	184.0 (163.0–208.0)	180.0 (159.2–186.5)	0.117
HDL-c (mg.dl^−1^)^*∗∗*^	50.0 (41.0–56.0)	50.0 (42.8–57.0)	0.639
LDL-c (mg.dl^−1^)^*∗∗*^	109.0 (87.0–131.0)	104.5 (83.0–115.5)	0.084
TG (mg.dl^−1^)^*∗∗*^	125.0 (97.0–152.0)	129.0 (91.8–148.2)	0.988
C-RP (mg.dl^−1^)^*∗∗*^	0.4 (0.4–0.4)	0.4 (0.4–0.7)	0.511
HbA1c (mg.dl^−1^)^*∗∗*^	5.7 (5.5–6.1)	5.8 (5.5–6.2)	0.954
IFN*γ* (pg/ml)^*∗∗*^	5.9 (5.4–6.6)	5.5 (4.9–6.5)	0.283
TNF*α* (pg/ml)^*∗∗*^	1.7 (1.1–2.4)	1.7 (1.0–2.6)	0.987
IL-10 (pg/ml)^*∗∗*^	3.4 (2.9–4.0)	3.3 (2.8–3.8)	0.615
IL-6 (pg/ml)^*∗∗*^	4.7 (3.5–8.8)	6.4 (3.8–12.8)	0.350
IL-4 (pg/ml)^*∗∗*^	1.7 (1.3–2.1)	1.6 (1.0–1.9)	0.192
IL-2 (pg/ml)^*∗∗*^	9.0 (8.6–9.9)	9.3 (8.6–9.8)	0.546
Grip strength (kgf)^*∗∗*^	18.6 (14.0–24.6)	16.1 (13.6–18.7)	0.041
Walking time (s)^*∗∗*^	5.0 (4.3–6.7)	7.0 (5.6–8.8)	0.001
Current diabetes (%)^†^	27.0	40.0	0.173
Current SAH (%)^†^	80.2	87.5	0.347
Current osteoporosis (%)^†^	44.0	39.3	0.527
Current arthritis (%)^†^	32.2	30.8	0.622
Current depression (%)^†^	29.5	25.9	0.685
History of cancer (%)^†^	16.7	21.4	0.546

BMI = body mass index, C-RP = C-reactive protein, HC = hip circumference, MMSE = Mini Mental State Examination, TC = total cholesterol, TG = triglycerides, HbA1c = glycated hemoglobin A1c, *SAH = systemic arterial hypertension*, WC = waist circumference, and W-H = waist-to-hip. ^*∗*^*t*-test for independent samples, *data as mean and standard deviation.*^*∗∗*^Nonparametric test of Mann–Whitney, *data as median and interquartile range*. ^†^Chi-square test, *data as within-group proportions*.

**Table 2:** Correlation analysis of the *immune mediators* with anthropometric and metabolic characteristics in a *whole-group approach* (*n* = 161).

	Log-transformed scores					
	TNF*α*	IFN*γ*	IL-10	IL-6	IL-4	IL-2
BMI (kg.m^−2^)	0.116; 0.179	−0.051; 0.549	-0.065; 0.442	0.025; 0.774	0.141; 0.092	−0.035; 0.658
Body fat (%)	0.168; 0.051	−0.057; 0.503	0.070; 0.405	0.033; 0.702	0.078; 0.354	−0.036; 0.656
W-H ratio (cm)	0.137; 0.128	−0.058; 0.508	−0.157; 0.072	0.184; 0.038^*∗*^	0.152; 0.081	−0.051; 0.543
Glucose (mg/dL)	−0.067; .439	0.072; 0.390	−0.054; .519	−0.212; 0.012^*∗*^	−0.045; 0.593	0.066; 0.405
TC (mg.dl^−1^)	−0.043; 0.619	−0.118; 0.162	−0.080; 0.344	0.068; 0.427	−0.012; 0.888	−0.153; 0.053
HDL-c (mg.dl^−1^)	−0.115; 0.181	−0.047; 0.581	0.068; 0.421	−0.157; 0.066	−0.050; 0.556	−0.071; 0.371
LDL-c (mg.dl^−1^)	−0.024; 0.784	−0.063; 0.455	−0.072; 0.390	0.106; 0.215	0.009; 0.917	−0.132; 0.094
TG (mg.dl^−1^)	0.085; 0.323	−0.127; 0.130	−0.129; 0.124	0.073; 0.396	−0.016; 0.854	−0.075; 0.347
C-RP (mg.dl^−1^)	−0.008; 0.931	0.058; 0.510	0.096; 0.272	0.223; 0.011^*∗*^	0.024; 0.786	−0.035; 0.675
HbA1c (mg.dl^−1^)	0.029; 0.760	0.008; 0.925	−0.038; 0.650	−0.067; 0.432	0.026; 0.759	0.005; 0.905
AM (qty)	−0.031; 0.742	−0.051; 0.579	0.000; 0.997	-0.038; 0.687	-043; 0.640	0.010; 0.905

The Pearson correlation tests were used. Data are expressed in correlation index and significance level (*r*; *P*). ^*∗*^The correlation is significant in the level of 0.05. AM = amount of medicines, BMI = body mass index, C-RP = C-reactive protein, HbA1c = glycated hemoglobin A1c, TC = total cholesterol, TG = triglycerides, and W-H = waist-to-hip.

**Table 3:** Univariate binary logistic regression analyses in a *whole-group approach* (*n* = 161).

	Frailty component					
	Low grip strength			High walk time		
	OR	CI (95%)	*p*	OR	CI (95%)	*p*
W-H ratio (cm)	1.47	0.04–46.54	0.825	5.88	0.04–726.99	0.471
Glucose (mg/dL)	1.01	0.99–1.03	0.254	0.98	0.96–1.00	0.221
C-RP (mg.dl^−1^)	0.69	0.46–1,03	0.070	1.10	0.73–1.65	0.646
Log IL-6	4.68	1.59–13.78	0.005	2.29	0.87–6.04	0.093

CI = confidence interval, C-RP = C-reactive protein, log = logarithmically transformed, and OR = odds ratio.

## Data Availability

The data supporting the current study can be made available from the corresponding author upon reasonable request.
